# Optimizing the frequency of ecological momentary assessments using signal processing

**DOI:** 10.1017/S003329172510264X

**Published:** 2025-11-25

**Authors:** Hamidreza Jamalabadi, Tahmineh A. Koosha, Elina Stocker, Andreas Jansen, Ulrich W. Ebner-Priemer, Ricarda K.K. Proppert, Carlotta L. Rieble, Rayyan Tutunji, Eiko I. Fried

**Affiliations:** 1 Department of Psychiatry and Psychotherapy, Marburg University, Marburg, Germany; 2 Center for Mind, Brain, and Behavior (CMBB), Marburg University, Marburg, Germany; 3 Faculty of Medicine, University of British Columbia, Canada; 4 Core-Facility Brainimagin, Faculty of Medicine, Marburg University, Marburg, Germany; 5 Mental health Lab, Institute of Sports and Sports Science, Karlsruhe Institute of Technology, Germany; 6 Faculty of Social Sciences, Institute of Psychology, Leiden University, Netherlands

**Keywords:** ecological momentary assessment (EMA), major depression disorder (MDD), sampling rate, signal processing

## Abstract

**Background:**

Ecological momentary assessment (EMA) is increasingly recognized as a vital tool for tracking the fluctuating nature of mental states and symptoms in psychiatric research. However, determining the optimal sampling rate – that is, deciding how often participants should be queried to report their symptoms – remains a significant challenge. To address this issue, our study utilizes the Nyquist–Shannon theorem from signal processing, which establishes that any sampling rate more than twice the highest frequency component of a signal is adequate.

**Methods:**

We applied the Nyquist–Shannon theorem to analyze two EMA datasets on depressive symptoms, encompassing a combined total of 35,452 data points collected over periods ranging from 30 to 90 days per individual.

**Results:**

Our analysis of both datasets suggests that the most effective sampling strategy involves measurements at least every other week. We find that measurements at higher frequencies provide valuable and consistent information across both datasets, with significant peaks at weekly and daily intervals.

**Conclusions:**

Ideal frequency for measurements remains largely consistent, regardless of the specific symptoms used to estimate depression severity. For conditions in which abrupt or transient symptom dynamics are expected, such as during treatment, more frequent data collection is recommended. However, for regular monitoring, weekly assessments of depressive symptoms may be sufficient. We discuss the implications of our findings for EMA study optimization, address our study’s limitations, and outline directions for future research.

## Introduction

Major depressive disorder (MDD) is a prevalent and debilitating mental health condition that imposes significant economic and social burdens globally (Greenberg et al., 2015). Characteristically, symptoms of depression are not static (Cramer et al., 2016; Hofmann et al., 2016); they evolve and fluctuate over time, often influenced by a variety of environmental, psychological, and physiological factors. This necessitates a nuanced understanding and monitoring approach, which is where ecological momentary assessment (EMA) becomes indispensable (Trull & Ebner-Priemer, 2020). EMA provides a powerful tool to capture the ebb and flow of depressive symptoms in real time, offering insights into their variability and patterns over time (Hahn et al., 2021; Myin-Germeys et al., 2024; Trull & Ebner-Priemer, 2009). This method enables capturing moment-to-moment changes (aan het Rot et al., 2012) as they unfold and minimizes recall bias (Shiffman et al., 2008). This is crucial to facilitate an understanding of the temporal dynamics of MDD, enabling clinicians and researchers to identify patterns, triggers, and fluctuations in symptoms that might otherwise be overlooked (Bell et al., 2017; Fechtelpeter et al., 2023).

**Figure 1.**
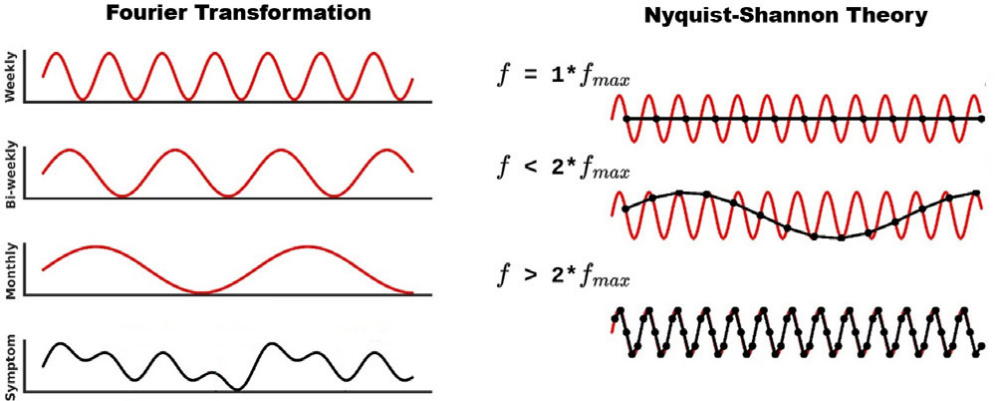
Nyquist–Shannon theorem and its use to optimize EMA sampling frequency. The left panel demonstrates the Fourier Transformation (Oppenheim et al., 1997), which converts a time-domain signal into its constituent frequencies within the frequency domain, with a focus on identifying the signal’s maximum (i.e. fastest) frequency component ( 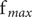 ). In this example, the depicted symptom (shown as a black signal) results from the combined effects of monthly, biweekly, and weekly fluctuations (shown as red signals), with the weekly fluctuations representing the fastest component. The right panel visualizes the sampling problem as described by the Nyquist–Shannon theorem, showcasing three different sampling rates ranging from equal to the maximum frequency ( 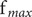 ) up to more than twice the maximum frequency (2 × 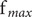 ). The aliasing effect occurs when the sampling frequency is less than 2 × 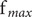 , causing different signals to become indistinguishable (aliased) from one another, as shown by the distorted black signals in the second and third subpanels on the right. According to the Nyquist–Shannon theorem, any sampling rate above (but not equal to) 2 × 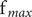 prevents aliasing and accurately represents the original signal.

However, as the adoption of EMA in psychiatric research continues to expand (Trull & Ebner-Priemer, 2020), a pressing question emerges: What is the ideal sampling rate for assessing depressive symptoms? The present authors have participated in numerous conferences on the topic of EMA over the last half-decade, and this question is one of the most commonly discussed topics. This is because the choice of sampling frequency not only has an impact on participant burden but can also have profound implications regarding the validity and reliability of EMA findings (Heron et al., 2017; White et al., 2023). On one extreme, a sampling rate that is too low may obscure true symptom patterns, creating ‘aliases’ – misleading artifactual patterns in the data (Oppenheim et al., 1997) (see Figure 1). On the other extreme, overly frequent sampling can overwhelm participants, either leading to generally reduced compliance and smaller sample sizes or to risk of bias because vulnerable populations may drop out of studies selectively. In either scenario, there is a risk of misinterpretation of the data. We note that while the remainder of this article focuses on depressive symptom dynamics as an exemplar construct, we believe the topic (and our proposed solution) is just as pressing and relevant for other areas of EMA research.

We address this challenge through a foundational result in signal processing: the Nyquist–Shannon theorem (Marks, 2012). This theorem establishes the minimum sampling frequency needed to accurately capture the fluctuations in any signal (i.e. measured variable). Simply put, the Nyquist–Shannon theorem states that the sampling rate should be greater than twice the highest major frequency component present in the data. This approach is crucial for ensuring that all the original signal’s information is captured, thereby avoiding ‘aliasing’. A notable application of the Nyquist–Shannon theorem can be seen in neuroscience, particularly in determining the optimal number of EEG channels on the head (Srinivasan et al., 1998), as well as in setting the appropriate temporal frequency for measurements in fMRI and EEG studies (Giannelli et al., 2010; Huotari et al., 2019). Beyond neuroscience, the Nyquist–Shannon theorem is a cornerstone of measurement theories across various fields, such as engineering and physics (Vaidyanathan, 2001). This underscores its fundamental importance in accurately capturing and analyzing complex signals in a wide range of scientific disciplines (Kanatsoulis et al., 2019; Srinivasan et al., 1998).

In the context of EMA research in mental health, constructs with a high temporal resolution such as mood, anhedonia, or fatigue can be conceptualized as latent continuous processes that evolve over time in response to internal states and external influences. While EMA captures these processes via discrete self-reports, typically using Likert or visual analog scale (VAS) at specific time points, it provides snapshots of an underlying dynamic trajectory. This conceptualization allows us to approximate EMA data as a sampled version of a continuous-time signal. Importantly, therefore, although the Nyquist–Shannon theorem was originally developed for continuous, band-limited signals, this approximation enables us to apply its core insight: to faithfully reconstruct a signal without distortion (i.e. aliasing), it must be sampled at a rate greater than twice its highest frequency component. For example, if depressive symptoms fluctuate with monthly, biweekly, and weekly cycles (see Figure 1), the theorem implies that EMA measurements should occur at least twice per week to preserve the full range of symptom dynamics. While EMA data are discrete and constrained by scale resolution, the signal-processing framework remains valid under the assumption that the underlying psychological processes are continuous. This approach thus provides a principled, mathematically grounded method for determining optimal EMA sampling frequencies and minimizing information loss due to undersampling (Oppenheim et al., 1997).

In this study, we apply the Nyquist–Shannon theorem to infer the optimal sampling rate for depressive symptomatology in two independent datasets. Dataset 1 was collected as part of the WARN-D project (Fried et al., 2023) and includes daily 1–7 Likert assessments for 3 months from 368 participants using an adapted version of the Patient Health Questionnaire-2 (PHQ-2) (Löwe et al., 2005), which captures the two core symptoms of MDD according to the DSM-5 (American Psychiatric Association & Association, 2013). The large sample size and extended duration of this dataset provide a robust foundation for our analysis. Dataset 2, originally published by Fisher et al. (2017), offers a more granular view, with 0–100 VAS measurements taken four times daily for 1 month. It not only includes adapted PHQ-2 assessments but also covers a broader spectrum of depressive symptoms as defined in the DSM-5 (American Psychiatric Association & Association, 2013). The frequent measurements throughout the day allow us to address potential aliasing effects that might be present in the first dataset, particularly those related to circadian rhythms versus daily symptom fluctuations. For example, if Dataset 2 reveals relevant fluctuations in depressive symptoms throughout the day or on a daily level, these would point to potential aliases present in Dataset 1. The parallel investigation of these datasets thus offers a unique opportunity to apply and validate our methodology. By leveraging the strengths of both datasets, we aim to refine our understanding of the optimal rate for monitoring depressive symptoms, ensuring that our results are both robust and applicable across a range of assessment scenarios. Figure 1 outlines the rationale behind our methodology and the steps involved in our analysis.

## Methods

### Dataset 1: WARN-D

The WARN-D project aims to build an early warning system for depression. It is a 2-year prospective, multicohort online study. Briefly, WARN-D tracks around 2,000 students over a span of 2 years, divided into four cohorts. For participation in the study, students need to be at least 18 years old and study at a Dutch higher education institution. Exclusion criteria are, among others, moderate to severe levels of depression before commencing the study. A detailed list of inclusion and exclusion criteria, along with the rationale, objectives, methods, and measures, are provided in the WARN-D protocol paper (Fried et al., 2023).

In addition to other daily and weekly surveys of varying lengths, participants received a smartphone prompt via the Ethica app (https://avicennaresearch.com/) every evening sometime between 9.04 pm and 9.34 pm; the survey expired after 20 minutes. This survey was available in both Dutch and English (participants could choose their preferred language). It included two items we focus on here, tapping into the DSM-5 core symptoms of MDD: depressed mood (‘Today, I felt down or depressed’) and anhedonia (‘Today, I had little interest or pleasure in doing things’), answered on a 1–7 Likert scale ranging from ‘not at all’ to ‘very much.’ The items are similar to the PHQ-2 items that also capture DSM-5 core symptoms; differences lie in the time frame (the PHQ-2 is focused on the last 2 weeks) and question type (PHQ-2 asks to what degree symptoms bothered people).

The current analysis uses EMA data of Cohort 1 (*N* = 300) and Cohort 2 (*N* = 307) of WARN-D, collected between December 2021 and August 2022. A preregistered random sample of 70% of participants was available for analysis; the remaining 30% of WARN-D data are under embargo for future out-of-sample prediction analyses (https://osf.io/6ephu/). To ensure sufficient quality of data for the analyses, we excluded subjects with fewer than 20 consecutive days of measurements and gaps in assessments longer than 3 days. After these adjustments, the dataset was reduced to 368 participants with a total of 18,020 data points, averaging 48.83 ± 20.64 measurements per participant. Subsequently, missing values were addressed using linear interpolation, which increased the total number of data points to 21,352, corresponding to an average of 57.79 ± 21.15 measurements per participant. See the section on robustness analyses for other ways in which we imputed data to ensure results are not due to a specific imputation technique.

The WARN-D study is funded by the European Research Council under the European Union’s Horizon 2020 research and innovation program (No. 949059); the data collection was approved by the Leiden University Research Ethics Committee Leiden (2021-09-06-E.I.Fried-V2-3406), and the study was exempted from having to obtain ethics approval under the Medical Research Involving Human Subjects Act. WARN-D data will be available on the WARN-D project hub (https://osf.io/frqdv/) once data collection of all cohorts is finalized, cleaned, and anonymized. The exact participant IDs used for this article can be found at https://osf.io/dtjnz/.

### Dataset 2

This study presents a post hoc analysis of data from Fisher et al. (2017). It is from a multiphase, personalized psychotherapy study focusing on individuals diagnosed with either primary generalized anxiety disorder (GAD) or primary MDD. Participants aged 18–65 years, owning a web-enabled mobile phone, and with a primary diagnosis of GAD or MDD were included. Those with a history of psychosis or mania, undergoing concurrent treatment, who had received cognitive behavioral therapy in the past year, or using PRN medication were excluded. A total of 40 participants were enrolled, and data are publicly available for analysis at https://osf.io/5ybxt/. Upon enrollment in the study, the mobile phone numbers of these 40 participants were registered in a secure web-based survey system between 30 and 40 days. This system sent out survey prompts four times daily via text messages, each containing a hyperlink to the survey. Participants used a 0–100 VAS to rate their experiences of various symptoms over the preceding hours. These items aligned with the DSM-5 symptom criteria for GAD and MDD (down and depressed, hopeless, loss of interest or pleasure, worthless or guilty, worried, restless, irritable, difficulty concentrating, muscle tension, fatigued). Additionally, participants rated 11 other items gauging positive affect (positive, energetic, enthusiastic, and content), negative affect (angry and afraid), rumination (dwelling on the past), behavioral avoidance (avoided people, avoided activities, and procrastinated), and reassurance seeking (sought reassurance). To operationalize the PHQ-2 in our analysis, we used items measuring depressed mood (‘To what degree have you felt down or depressed over the preceding hours’) and anhedonia (‘To what extent have experienced loss of interest or pleasure over the preceding hours’). In this study, we included data from participants who had at least 20 consecutive days of measurements, allowing for up to three consecutive days of missing data. This filtering process led to a final sample of 39 participants, totaling 4,411 data points, with an average of 33.82 ± 5.03 days of measurements per subject. To handle missing values, we applied linear interpolation, which increased the total number of data points to 5,536, corresponding to an average of 34 ± 5.17 days per participant.

### Power spectrum density of EMA data

In signal processing (Oppenheim et al., 1997), a discipline centered on analyzing signals such as time series, a fundamental result is the decomposition of a time-based signal into a series of periodic components, each characterized by its unique frequency (see Figure 1), through Fourier transformation. By employing this method, we can precisely understand how different frequencies contribute to the overall behavior of a time series. This is often done by examining the power spectrum density (PSD) analysis (Oppenheim et al., 1997), which quantifies the contribution of each frequency component by estimating the area under the logarithmically scaled representation of the signal in the frequency domain (Oppenheim et al., 1997).

When PSD is applied to EMA data related to depressive symptoms, it allows us to see the distribution of power across various frequencies in the EMA data, providing insights into the relative importance of these frequencies within the total spectrum of our data. ‘Power’ in this context refers to the magnitude of variation or fluctuations within a specific frequency component, and it is estimated from the Fourier transformation of the data. In this article, to estimate the Fourier transformation of the data, that is, to transform the EMA data from the time domain to the frequency domain, we employed Welch’s method (Welch, 1967). This method is particularly adept at minimizing noise in the resulting power spectra (Hayes, 1996). It achieves this by segmenting the time-series data into overlapping parts, calculating a modified periodogram for each, and then averaging these periodograms. We used segments with 50% overlap, which is the most common implementation of Welch’s method (Rahi & Mehra, 2014). To visualize the results and estimate the maximum frequency in the data based on the Nyquist–Shannon theorem, we computed the cumulative PSD. This approach aggregates power from the lowest to the highest detectable frequency in our data, offering a comprehensive view of power distribution as one moves from lower to higher frequencies. Cumulative PSD, therefore, spans frequencies between zero (corresponding to the average of the EMA time series), reaching up to the maximum frequency detectable in the data, which is half the original sampling rate (i.e. every 2 days in Dataset 1 and every half-day in Dataset 2). We estimated the cumulative PSD for each person separately and displayed the results on a logarithmic scale. Using the logarithmic scale is recommended because the power of different frequency components can vary over several orders of magnitude. This scale compresses the wide range of values into a more manageable and interpretable form, allowing both small and large values to be visualized simultaneously (Oppenheim et al., 1997). We present our results from the perspectives of both frequency and time (see Table 1). A frequency of 1 indicates a signal where a full fluctuation cycle occurs in 1 day (see, e.g. Figure 1). Accordingly, a frequency of 0.1 corresponds to a signal taking 10 days to complete a cycle, and a frequency of 2 means the cycle completes in half a day. All analyses were conducted using Python 3.7. Additionally, the code used for this research is publicly accessible at our GitHub repository: https://osf.io/dtjnz/.

**Table 1.** Interpretation of power spectrum frequencies and implications for EMA sampling design

Frequency (1/day)	Corresponding period (days)	Description of fluctuation regime	Minimum EMA sampling rate required
0.03	33 days	Slow fluctuations (monthly cycle)	≥Every 16 days
0.07	14 days	Biweekly fluctuation	≥Every 7 days
0.1	10 days	Regular cyclical shifts	≥Every 5 days
0.5	2 days	Fast symptom changes	≥Daily
1.0	1 day	Daily rhythm	≥Twice daily
2.0	0.5 days (12 hours)	Within-day/circadian effects	≥Four times daily

### Analysis of symptom count impact on sampling frequency

To determine if incorporating a broader range of symptoms affects the required sampling frequency for assessing depression severity in EMA, we utilized Dataset 2, which includes high-density longitudinal measurements of 21 symptoms. We expanded the PSD analysis (see section ‘Power spectrum density of EMA data’) to include an incremental number of symptoms to explore whether a more comprehensive set of depression items influences the necessary sampling frequency. We repeated the PSD analysis for groups of 2, 5, 9, 14, and 21 symptoms randomly selected from the 21-item questionnaire available from data Dataset 2. The symptoms analyzed included ‘procrastination’, ‘rumination’, and feelings of being ‘energetic’, ‘worried’, ‘fatigued’, ‘positive’, ‘hopeless’, ‘reassured’, ‘concentrated’, ‘restless’, ‘content’, ‘angry’, ‘enthusiastic’, ‘afraid’, ‘tense’, ‘guilty’, ‘irritable’, along with tendencies to ‘avoid people’ and ‘avoid activities’.

To assess the impact of varying item counts on the PSD, we focused on the slope of the cumulative PSD as a function of frequency. This method measures how quickly the cumulative PSD approaches its maximum value (100%). A change in slope indicates a difference in the power distribution between low and high-frequency components. We estimated the slope by fitting a linear regression to the log–log plot of cumulative PSD for frequencies above faster than 1/30.

### Robustness analyses

To ensure the results stayed the same for different preprocessing of the data, we tested the impact of analytic choices (imputations, number of days) by repeating the process with different factors (see Supplementary Materials). Results remained robust.

## Results

### Dataset 1

**Figure 2.**
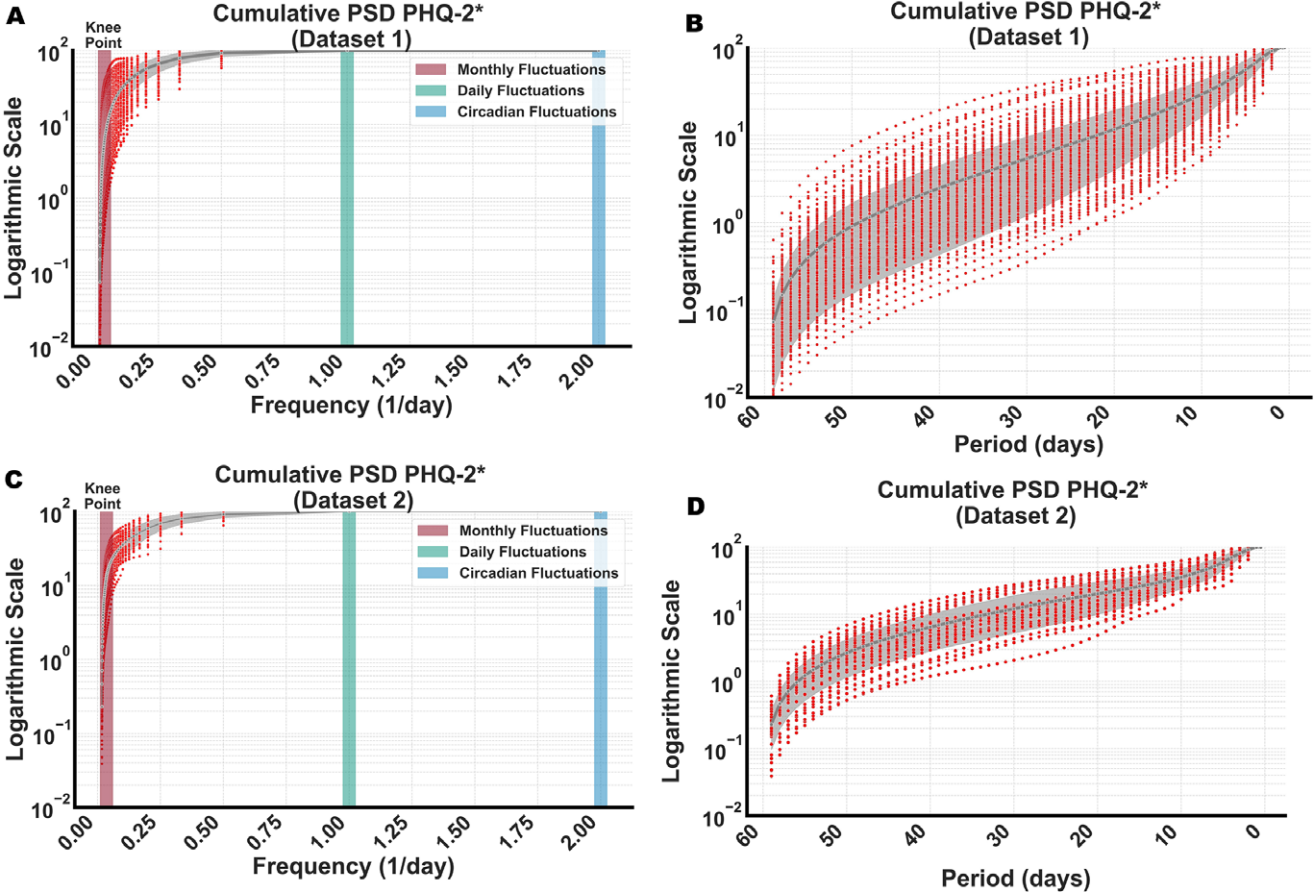
Cumulative PSD of depressive symptoms on a logarithmic scale. This figure illustrates the distribution of spectral power across various frequency components, which is critical for determining the optimal sampling frequencies for monitoring depressive symptoms. According to the Nyquist–Shannon theorem, the ideal sampling rate should be at least twice the fastest frequency that holds significance in these analyses. Given that our data comprises estimates of PHQ-2 scores (our data are measured once a day or four times a day, while the original PHQ-2 asks about the past 2 weeks), we denote the measured variable as PHQ-2* to reflect its estimated nature. (A) Cumulative spectral power of PHQ-2 in Data 1, displayed by frequency (1/day) – see Table 1 for interpretation. A frequency of 0.5 corresponds to fluctuations that repeat every second day or less frequently, and a frequency of 0.1 to those occurring every 10 days or less frequently – see Table 1 for interpretation. (B) Cumulative spectral power of PHQ-2 in Data 1, shown by time (days). A time of 2 days illustrates cumulative PSD for fluctuations that repeat every 2 days and slower. (C) Cumulative PSD for Data 2, presented by frequency (1/day). (D) Cumulative PSD for Data 2, shown by time. Red dots represent the PSD data of individual participants (a random selection of n = 39 in Dataset 1, all n = 39 in Dataset 2), while the gray line indicates the mean value.

We first focused on examining the EMA sampling properties of Dataset 1. Our approach involved estimating the cumulative PSD for each participant’s depressive symptomatology, as measured by the average of two items in the adapted PHQ-2. We then averaged these individual estimates to derive normative values (Figure 2A), which are presented on a logarithmic scale ranging from 0 to 100 %. Given that the EMA data was collected daily, our analysis of cumulative PSD extended from frequencies of f = 0 (corresponding to the long-term mean symptom level) to f = every 2 days (corresponding to symptom fluctuations that complete every 2 days). This is because components of the signal that have frequencies higher than the Nyquist–Shannon rate (i.e. twice the highest frequency component, which is every 2 days in Dataset 1) will be aliased back into the frequency range below every Nyquist–Shannon frequency, creating ambiguity about the true frequencies present in the sampled signal (Oppenheim et al., 1997) (see Figure 1).

Our results (see Figure 2A) indicate a knee point in the cumulative PSD at frequencies around 0.03 per day, which corresponds to monthly fluctuations. From a signal-processing standpoint, therefore, measuring at twice this frequency – approximately 0.06 per day or every 16 days – represents an optimal trade-off (see Table 1): this balance maximizes the increase in cumulative PSD gained from more frequent measurements while maintaining meaningful data increments. It is important to note that we relied on visual inspection to determine the frequency of the knee point (also called elbow point). Different algorithms could offer varying estimations depending on their underlying hypotheses (Satopaa et al., 2011). However, in the absence of any prior hypothesis regarding the characteristics of the knee point, visual inspection remains our method of choice.

**Figure 3.**
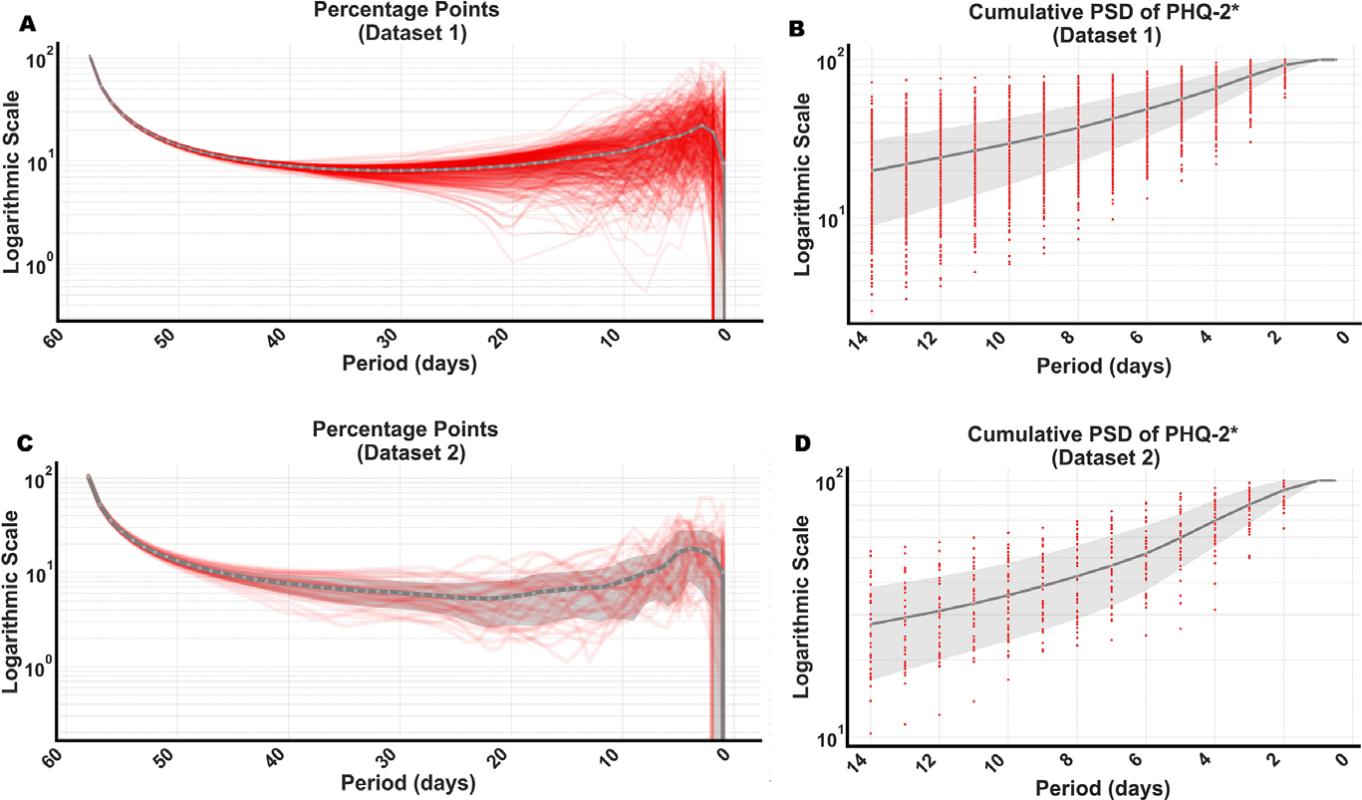
Identification of high-frequency components in cumulative PSD. This figure highlights changes in cumulative PSD by examining point percentage changes. These changes are calculated by estimating the difference in cumulative PSD from one frequency to the next, where an increasing pattern or a peak suggests significant new dynamics in the underlying EMA data. Given that our data comprises estimates of PHQ-2 scores (our data are measured once a day or four times a day while the original PHQ-2 asks about the past 2 weeks), we denote the measured variable as PHQ-2* to reflect its estimated nature. (A) Point percentage changes for PHQ-2* in Dataset 1 show an increasing pattern, peaking at around every 14 days and more frequently at every 2 days. (B) Cumulative PSD of PHQ-2* in Dataset 1. At frequencies representing fluctuations occurring every 14 days or more frequently, there is an almost linear increase on the logarithmic scale, indicating a substantial rise in cumulative PSD that suggests the need for more frequent EMA sampling. (C) Point percentage changes for PHQ-2* in Dataset 2, following a similar pattern as in (A). (D) Cumulative PSD for Dataset 2, mirroring the trends observed in (B). Red lines represent the percentage point changes of individual participants (a random selection of n = 39 in Dataset 1, all n = 39 in Dataset 2), and red dots indicate their cumulative PSD. The gray line represents the mean value.

Beyond this frequency, the rate of increase in cumulative PSD begins to plateau, as shown in Figure 2A. However, a more detailed examination, as depicted in Figure 3A, reveals two distinctive shifts in this trend. Specifically, we analyzed the percentage changes in cumulative PSD, that is, the relative increase in cumulative PSD from one frequency to the next. Typically, we would expect these values to decline unless new dynamic patterns emerge, signaling changes in the underlying data. Initially, starting from bimonthly fluctuations (corresponding to components of symptoms changing at f = 1/60), the percentage changes in cumulative PSD decrease. However, beyond the monthly frequency and particularly after fluctuations every 2 weeks (corresponding to components of symptoms changing at f = 1/14), these changes start to increase again, peaking notably at a frequency close to 2 (indicating symptom changes approximately every 2 days, i.e. f = 1/2). To further highlight these variations, we magnified the cumulative PSD in the range where symptom fluctuations cycle from every 14 days to every 2 days (Figure 3B), observing (employing visual inspection) a sharp increase.

Taken together, these findings suggest that the optimal sampling rate for EMA studies depends on the regime deemed ‘sufficient’ for explaining the cumulative PSD (see section Discussion). In line with the Nyquist–Shannon theorem, which recommends that the optimal frequency is more than twice the highest meaningful frequency, our results thus imply that an effective EMA strategy could involve measurements as frequently as every 2 weeks, every week, or daily, effectively doubling the frequencies of the monthly, biweekly, and 2-days benchmarks we identified, respectively.

### Dataset 2

Unlike Dataset 1, the sampling frequency in Dataset 2 was four times daily, allowing us to extend our estimation of the cumulative PSD to frequencies as frequent as every 0.5 days. Despite the increased frequency, a similar pattern emerged, as illustrated in Figures 2 and 3. Notably, a more distinct peak at the frequencies of every 2 days is visible in Figure 3B compared to Figure 3A, implying a distinctive peak in symptom fluctuation frequency every second day.

Further, comparing the results between the two datasets, a few key observations stand out. First, the results closely align with each other, showing a strong correlation between the curves in Figures 2 and 3 between two datasets (r > 0.95). Second, there is a notable increase in the variability across subjects in Dataset 2 for point percentage changes in cumulative PSD (i.e. the variance across subjects in the percentage point increases, Figure 3A vs. Figure 3B) concerning symptom fluctuations occurring every 26–18 days (see Table 1). This increase is statistically significant, with Bonferroni-corrected Levene’s test p-values ranging between 0.006 and 0.038. This indicates that the healthy and subclinical participants in Dataset 1 exhibit more uniform behavior in terms of symptom fluctuations at medium frequencies (cycles between 18 and 26 days) compared to the participants in Dataset 2, who were diagnosed with MDD, GAD, or both. Third, there is a small but consistent difference in cumulative PSD between Datasets 1 and 2, starting at very low frequencies (see Supplementary Material Figure S1 for a clear comparison), which indicates an aliasing effect from higher to lower frequencies (see Figure 1B).

### Inclusion of more symptoms

Finally, we tested whether incorporating a broader range of symptoms would impact the required sampling frequency for assessing depression severity in EMA. The cumulative PSD of the EMA data from Dataset 2 using an increasing number of symptoms and compared the slopes of the cumulative PSD to determine if adding more symptoms changes the necessary sampling frequency (see Methods for details). A flatter slope suggests a smaller contribution from higher-frequency components, indicating slower symptom fluctuations, while a steeper slope suggests a greater contribution from high-frequency components, indicating more rapid symptom fluctuations. Our analysis indicated that the slopes were consistent across different symptom counts, ranging from 0.96 to 0.98, with no significant differences found in pairwise comparisons of these data (see Supplementary Material Figure S2). Visual inspection of these results also demonstrates that the cumulative PSD across all datasets is quite similar. These findings suggest that increasing the number of symptoms used to estimate depression severity does not impact the cumulative PSD, and therefore does not affect the optimal EMA sampling rate.

## Discussion

Recognizing that fluctuations in symptoms and mood are key characteristics of depressive disorders, a longstanding question has been: What is the optimal frequency for measuring EMA, like those tracking depressive symptoms? In our study, we used a well-established, theory-driven approach rooted in signal processing to explore this issue in mental health data. We first briefly reiterate the findings before we move on to a discussion of other factors that play a role in determining optimal sampling frequency.

Importantly, our analysis revealed a consistent pattern across both datasets: symptomatic fluctuations predominantly occur at a slower pace. This observation supports the rationale behind routine diagnostic instruments, where symptoms are commonly queried for 2-week periods (Beck et al., 1996; Hamilton, 1960; Montgomery & Åsberg, 1979). Notably, we identified distinctive shifts in symptom patterns at intervals of monthly, biweekly, and every other day, potentially indicating that the optimal measurement strategy chosen – twice faster than these scales – should align with the particular study’s purpose. When we analyzed Dataset 2 with a much higher assessment frequency (four times daily) than Dataset 1 (once daily), we observed similar knee points in the PSD. Additionally, a comparison of both PSD graphs showed only a slight difference in the cumulative PSD between the two datasets, indicating minimal aliasing when measurements are taken once compared to four times daily. We showed that these results are consistently observed across subjects, datasets, and measurement questionnaires, regardless of the number of items they contain.

Beyond assessment and prediction, our methodology may also inform explanatory research by providing a way to disentangle different sources of variability in symptom trajectories and the mechanism of change (Heshmati et al., 2024). PSD analysis decomposes EMA time series into frequency components, separating slow dynamics (e.g. multiweek or monthly shifts) from faster fluctuations (e.g. daily or within-day changes). These temporal regimes may reflect different underlying processes: slower cycles could be linked to stable individual factors such as trait vulnerabilities, illness phase, or long-term environmental changes, while faster cycles may capture transient contextual influences such as acute stressors, social events, or daily routines. By quantifying where the spectral power is concentrated for each individual, PSD enables researchers to (a) detect which temporal scales dominate a person’s symptom dynamics, (b) compare these scales across individuals or subgroups, and (c) match sampling rates to the relevant mechanisms of interest. These insights can support modeling approaches – such as vector autoregression to assess symptom–context interactions (Epskamp et al., 2018) or dynamical systems frameworks that identify transitions and tipping points in mental states (Fechtelpeter et al., 2023; Scheffer et al., 2024).

### Optimal EMA sampling rate

These findings are both straightforward and interpretable, yet they come with a set of important practical and theoretical considerations we discuss next. For instance, the observed interindividual variability in PSD, particularly the higher variance in clinical populations, suggests that personalized sampling schedules may enable more idiographic modeling of mechanisms such as treatment responsiveness. This leads us to an important question: What are the relevant frequency components in the EMA of depression?

Our results suggest that EMA data for depressive symptoms is not inherently band-limited, meaning that, in principle, higher sampling frequencies could capture additional signal power without theoretical upper bounds on useful information. However, there is a practical point of diminishing returns where further increases in sampling frequency become clinically irrelevant, as they may not meaningfully enhance insights into symptom dynamics while imposing unnecessary burden on participants. We find it helpful to draw a comparison to neuroscientific literature on EEG analysis. Most EEG studies focus on frequencies up to the beta-band, typically under 40 Hz, despite scalp EEG capturing details up to 100 Hz (Hosseini et al., 2020). This selection stems from a key insight: much like depressive symptoms, the bulk of signal power in EEG resides in lower frequencies. This renders higher frequencies less influential to, for example, shape EEG’s topological patterns (e.g. in microstates analysis, data up to 20 Hz are utilized; Sikka et al., 2020) although higher frequencies, such as those in gamma band, are still studied under certain paradigms (Fitzgerald & Watson, 2018).

In our analysis, we identified three distinct regimes in the frequency analysis of depressive symptoms: monthly, every other week, and every other day. This suggests that the most prevalent symptom changes likely manifest within these intervals. To make a final choice of EMA sampling rate, one has to understand what these frequencies represent and how they may shape the experience of depression. To guide selection, we propose two key perspectives.

First, in the context of episodes of MDD, focusing on the last 2 weeks is common because it aligns with how diagnostic interviews for MDD are carried out (Beck et al., 1996; Hamilton, 1960; Montgomery & Åsberg, 1979), implicitly acknowledging the critical role of these mid-range dynamics in depression. Moreover, while it has been possible to accurately predict depressive symptoms within a 2-week timeframe (Hahn et al., 2021; Jamalabadi et al., 2022), extending these predictions to longer periods remains a challenge. Further, we observed a notable difference between the cumulative PSD of Dataset 1 with healthier participants than Dataset 2 (patients with depression or anxiety) in terms of cross-participant variance, particularly in slower symptom frequencies changing every 18–26 days. Together, these observations highlight the importance of incorporating these mid-range and low-frequency insights, over extended periods, into both research and clinical settings. This may relate to the episodic nature of MDD, characterized by episodes that can last many months (Spijker et al., 2002).

Second, for tracking rapid changes, such as responses to environmental stressors, treatment effects, or daily fluctuations (Bucur et al., 2024), higher-frequency regimes (e.g. daily or multiple times per day) may be warranted to capture abrupt shifts.

Practically, the knee-point in the cumulative PSD curve serves as a practical heuristic for identifying this transition from clinically relevant to irrelevant frequencies, where additional sampling yields minimal incremental power. While visual inspection is a valid starting point, we acknowledge its subjectivity and recommend objective criteria for enhanced reproducibility: define the knee as the frequency where cumulative PSD reaches a predefined threshold. This threshold can be algorithmically determined using methods like the Kneedle algorithm (Satopaa et al., 2011) or second-derivative maximization on the PSD curve. Such criteria have proven effective in similar applications (e.g. signal compression in biomedical engineering), and should be validated across clinical groups (e.g. MDD versus anxiety disorders) and conditions (e.g. stable versus acute phases) to ensure robustness. In our analyses, these objective methods produced estimates highly consistent with our visual inspection, knee points around 0.03 ± 0.01 for Dataset 1 and 0.02 ± 0.01 for Dataset 2, reinforcing the robustness of our findings. Nevertheless, the small but significant differences between definitions highlight the need for further research on their clinical relevance in the context of EMA.

### Interindividual variability and personalized EMA schedules

Optimal frequencies should be tailored to the study’s aim and sample characteristics. Our analyses primarily rely on group-level PSD averages, which provide a general recommendation – for example, weekly sampling may be optimal for many applications, balancing the capture of mid-range fluctuations against participant burden. However, EMA data exhibit substantial interindividual variability, as evidenced by higher individual variability in Dataset 2’s clinical sample, particularly in slower frequencies. Relying solely on group averages risks overlooking meaningful differences: an ‘optimal’ frequency derived from averages might under-sample individuals with rapid fluctuations (e.g. those with high reactivity to stressors) or over-burden more stable individuals whose symptoms change slowly.

To estimate the optimal frequency for each group and condition, further analyses are required. Here, we provide a stepwise protocol that can be implemented using the codes and tools developed as part of this study. This procedure should be repeated for each new diagnosis and subgroup to generate optimal recommendations that go beyond the general guidelines presented here. The protocol involves: (1) collecting a short baseline period of high-frequency EMA data for each participant (e.g. two to four assessments per day over approximately 1 month) to capture multiple fluctuation regimes from circadian to multiweek cycles; (2) estimating the individual PSD (e.g. using Welch’s method) to quantify how signal variance is distributed across frequencies; (3) determining each person’s ‘knee point’ in the cumulative PSD using objective criteria such as the Kneedle algorithm, thereby identifying the lowest sampling rate that still captures the majority of variability; (4) validating the derived rate over a follow-up period by comparing reconstructed symptom trajectories against the baseline; and (5) conducting subgroup analyses – such as stratifying by severity level, diagnosis, or comorbidity – to inform refined, population-specific recommendations.

### Limitations

Our study provides valuable insights, yet it is essential to acknowledge its limitations for a thorough understanding.

First, investigating the impact of the timing of measurements, whether regular or irregular, is crucial. For example, in Dataset 2, measurements were collected four times daily but not during the night, resulting in an inherently nonuniform sampling pattern. While the Nyquist–Shannon theorem applies to irregularly spaced measurements as well (Vaidyanathan, 2001), the presence of components like circadian rhythms or sleeping patterns may influence the PSD analysis and, consequently, the interpretations we make. From a mathematical perspective, small deviations from uniform sampling (so-called ‘jitters’) are tolerable, but only within strict limits. Theorems such as Kadec’s (Young, 1981), formalize these limits, showing that reconstruction remains stable only if irregularities are bounded. Importantly, this implies that simply achieving a sufficient number of samples ‘on average’ is not adequate if prolonged gaps occur; rather, the sampling strategy must be matched to the locally expected frequency content of the underlying process. In practical terms, for studies investigating rapid fluctuations (e.g. using random or event-contingent sampling), data collection schedules should be designed to capture the fastest-changing components. Future work should investigate methodological adjustments or alternative analytical frameworks to account for nonuniform sampling intervals in event-contingent designs and to more accurately characterize the influence of circadian and other time-dependent variations.

Second, questions remain regarding the generalizability of our results, related to both content and context. While we found strong convergence between datasets despite differences in measurement context and population, we observed higher cross-participant variability in Dataset 2 (which included individuals with clinical diagnoses; see Figure 3), pointing to potential differences in temporal dynamics. Further consideration concerns the framing of EMA items, which may also affect generalizability. Dataset 1 used end-of-day retrospective ratings (e.g. ‘Today, I felt…’), potentially acting as a low-pass filter that smooths intraday fluctuations and attenuates higher-frequency signal components. In contrast, Dataset 2 used momentary-style prompts referencing the ‘preceding hours’, more aligned with conventional EMA practices. Although this difference could theoretically influence the observed power spectrum, particularly in the higher-frequency range, our analyses showed strong alignment between datasets, with only minor aliasing effects and similarly shaped PSD curves (see Supplementary Figure S1). This suggests that the dominant temporal features of depressive symptoms, at least as captured by PHQ-2-derived measures, are robust to differences in item framing. Further, a critical consideration is the disaggregation of symptom-level dynamics from those of syndromes (Fried, 2022). Different symptom types such as physical (e.g. fatigue, sleep disturbance), cognitive (e.g. concentration problems), and affective (e.g. depressed mood) symptoms may follow distinct fluctuation patterns. For instance, physical symptoms may exhibit faster changes and be sensitive to circadian cycles, warranting higher-frequency sampling. Such differentiation is a valuable direction for future research, and can be investigated using multivariate analytical techniques, such as cross-spectral analysis or dynamic factor models, applied to high-resolution datasets. Identifying these temporal signatures can improve explanatory models and support the development of precision EMA protocols tailored to specific symptom dynamics.

Third, while the introduced methodology can deconstruct any time series into its sinusoidal components, it struggles with abrupt changes in the temporal dynamics of signals, such as transitions from a healthy to a depressed state (Fried, 2022; Scheffer et al., 2024). Fourier transformation reveals an infinite spectrum of frequencies within the signal, meaning no finite sampling rate can fully capture these sudden shifts. This is not a critical limitation. The analytic method would replace a sharp step in a signal (e.g. a large abrupt change of the symptoms) with a slightly rounded one, which may still convey the essential information about the transition.

Finally, our study did not take into account external factors such as medication intake and alcohol consumption, which could influence the temporal dynamics of depressive symptoms on faster time scales than those we measured. These factors might also lead to significant but transient fluctuations in symptoms. If such factors are found to significantly impact symptom dynamics, they could necessitate more frequent sampling than our study suggests.

## Supporting information

Jamalabadi et al. supplementary material Jamalabadi et al. supplementary material

## Data Availability

All codes are publicly available at https://osf.io/dtjnz/. Raw Dataset 2 is available at https://osf.io/5ybxt/. Collection of the WARN-D data (Dataset 1) is still ongoing, and we want to avoid having different small parts of the data shared across many different papers. We will make data available on the WARN-D project hub (https://osf.io/frqdv/) when all data are collected, checked, and cleaned (excluding potentially identifiable data). To make the present analysis reproducible in the future, we share the exact participant IDs we used for this article in the supplementary materials and at https://osf.io/dtjnz/.

## Supplementary material

The supplementary material for this article can be found at http://doi.org/10.1017/S003329172510264X.
