# Supplementary material for: Optimizing the frequency of ecological momentary assessments using signal processing
Source: Psychol Med. 2025 Nov 25;55:e358. doi: 10.1017/S003329172510264X (PMC12671912; doi:10.1017/S003329172510264X)
Supplement: Jamalabadi et al. supplementary material [file S003329172510264Xsup001.docx]

# Supplementary Materials

## Comparison of both datasets


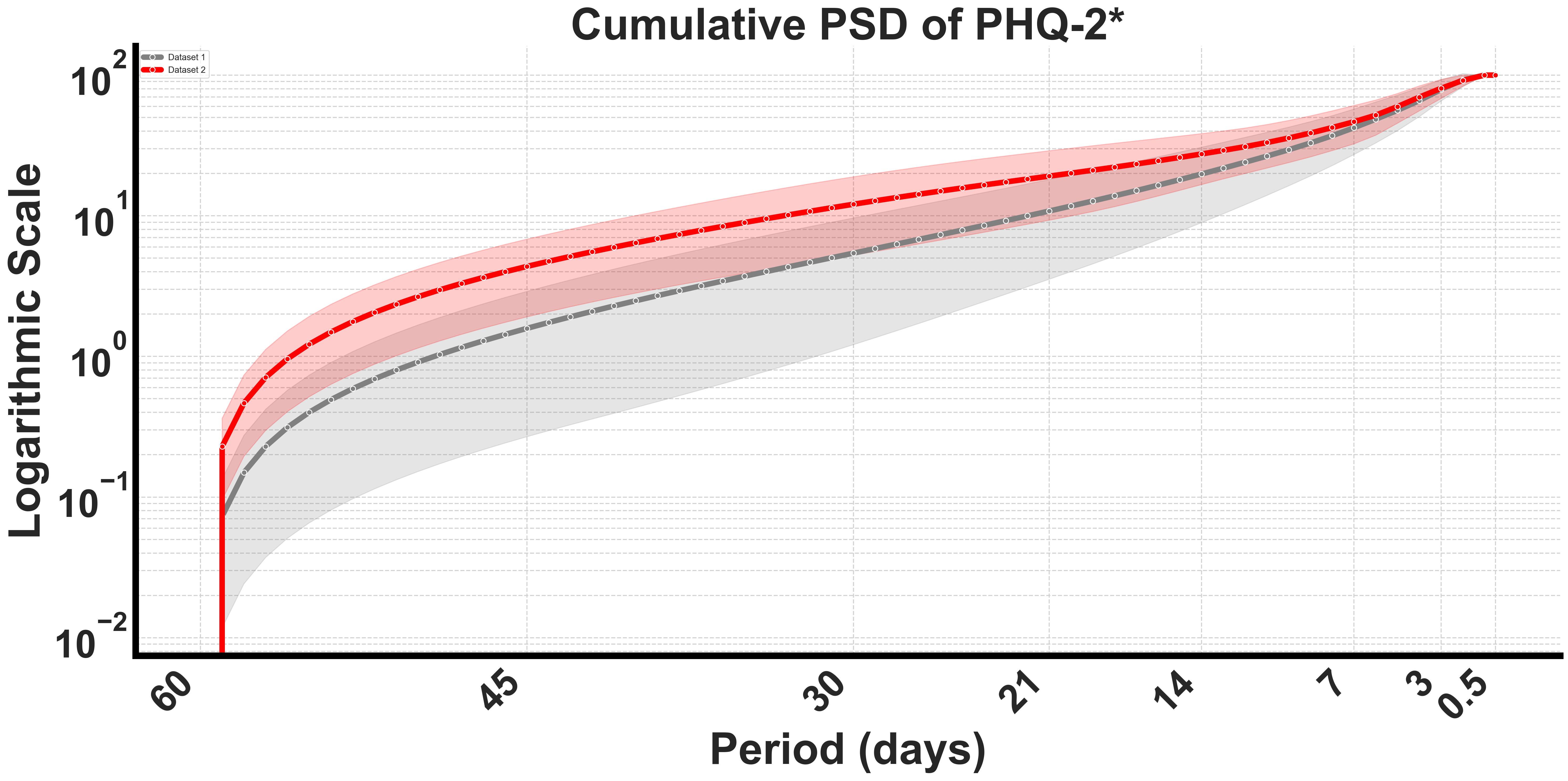


***Figure S1. Cumulative PSD of Depressive Symptoms on a Logarithmic Scale.*** *This figure displays the distribution of spectral power across various frequency components for both datasets. Solid lines represent the average cumulative for subjects in dataset 1 and dataset 2. The shaded areas indicate the mean error margins.*

## Effect of additional symptoms on cumulative PSD of depression severity scores


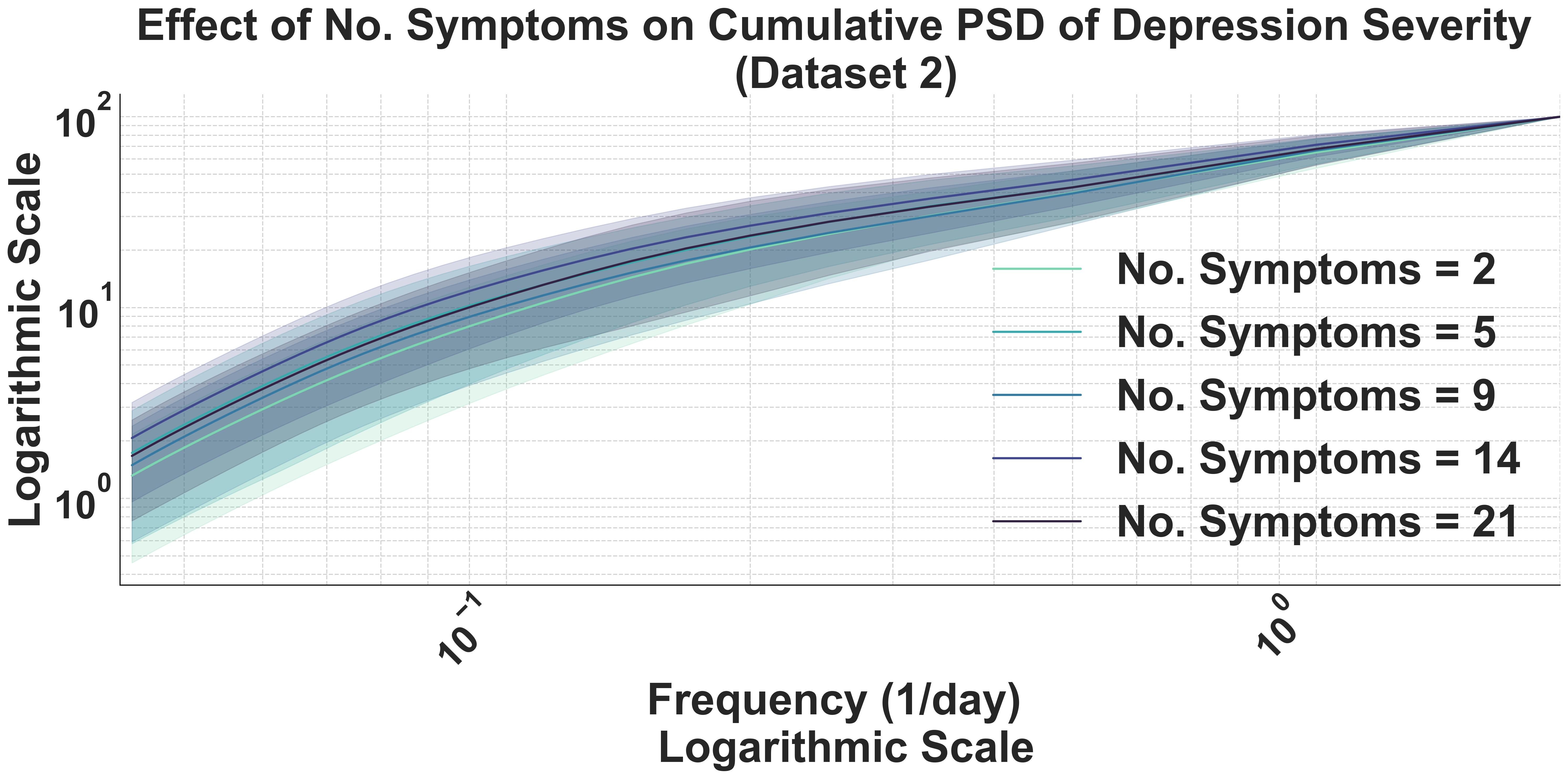


***Figure S2. Cumulative PSD of Depression Severity Scores Using Different Numbers of Items.*** *This figure illustrates the distribution of spectral power across various frequency components in dataset 2, where daily measurements included 21 items. Solid lines represent the average cumulative PSD, and colored shadows indicate error margins. Our analysis reveals that the cumulative PSD for all estimations are quite similar, and statistical tests comparing their slopes show no significant differences.*

## Robustness analysis

To ensure the robustness of our findings, we conducted additional analyses to assess the impact of parameter choices on the estimated PSD curves presented in this paper. Specifically, we replicated the cumulative PSD as shown in Figure 2B and Figure 2C with variations in two key parameters:

1. The minimum number of consecutive measurement days required to estimate PSD.
2. The method for imputing missing data

**Effects of data length on the results.** For our primary analysis in the paper, we utilized data from participants who had at least 20 consecutive days of measurements. To verify that this selection criterion did not significantly influence our results, we conducted a replication analysis, as depicted in Figure S3. In this replication, we included two additional variations: one where data from participants with at least 15 consecutive days were analyzed, and another with data from those with at least 30 consecutive days. The analysis revealed (visual inspection) that the variations in results across these different datasets are negligible, indicating the robustness of our initial findings.

**Effects of data preparation on the results.** In our primary analysis presented in the paper, we employed a linear interpolation method to impute missing data. To ensure that our choice of imputation technique did not substantially impact our findings, we conducted a replication analysis, which is illustrated in Figure S3. In this analysis, we tested three different imputation methods for missing data: mean imputation, linear interpolation, and nearest neighbor interpolation (Van Buuren, 2018). With the mean imputation method, we filled missing data points using the average values within the closest three values across time for Dataset 1 (WARN-D) and within a day for Dataset 2.. The nearest neighbor interpolation method involved replacing missing entries with the first, non-missing value in time. Linear interpolation was applied to estimate missing values by creating a linear fit based on nearby data points. As demonstrated in Figure S3, the comparison of these imputation techniques (visual inspection) shows that their impact on the overall results is negligible. This consistency across methods highlights the robustness of our findings, regardless of the imputation strategy used.





***Figure S3. Replication of Figure 2 Using Various Imputation Strategies Across Different Data Sets.*** *This figure shows the cumulative PSD for depressive symptoms on a logarithmic scale for both dataset 1 and dataset 2. Each dataset is analyzed using three different imputation techniques, represented by distinct colors in each panel. Since the data consist only of estimated PHQ-2 scores, reflecting symptoms from the previous day and the past few hours respectively, these scores are denoted as PHQ-2*.*

**Reference**

Van Buuren, S. (2018). *Flexible imputation of missing data*. CRC press.
